# Increased abundance of translation machinery in stem cell–derived neural progenitor cells from four schizophrenia patients

**DOI:** 10.1038/tp.2015.118

**Published:** 2015-10-20

**Authors:** A Topol, J A English, E Flaherty, P Rajarajan, B J Hartley, S Gupta, F Desland, S Zhu, T Goff, L Friedman, J Rapoport, D Felsenfeld, G Cagney, A Mackay-Sim, J N Savas, B Aronow, G Fang, B Zhang, D Cotter, K J Brennand

**Affiliations:** 1 Department of Psychiatry, Icahn School of Medicine at Mount Sinai, New York, NY, USA; 2 Royal College of Surgeons in Ireland, Beaumont Hospital, Beaumont, Dublin, Ireland; 3 Department of Biomedical Informatics, Cincinnati Children's Hospital Medical Center, University of Cincinnati, Cincinnati, OH, USA; 4 Department of Genetics and Genomics, Icahn School of Medicine at Mount Sinai, New York, NY, USA; 5 Childhood Psychiatry Branch, National Institute of Mental Health, National Institutes of Health, Bethesda, MD, USA; 6 Department of Developmental and Regenerative Biology, Icahn School of Medicine at Mount Sinai, New York, NY, USA; 7 UCD Conway Institute of Biomolecular and Biomedical Research, Dublin, Ireland; 8 Eskitis Institute for Drug Discovery, Griffith University, Brisbane, QLD, Australia; 9 Department of Chemical Physiology, The Scripps Research Institute, La Jolla, CA, USA; 10 Department of Neuroscience, Icahn School of Medicine at Mount Sinai, New York, NY, USA; 11 Friedman Brain Institute, Icahn School of Medicine at Mount Sinai, New York, NY, USA

## Abstract

The genetic and epigenetic factors contributing to risk for schizophrenia (SZ) remain unresolved. Here we demonstrate, for the first time, perturbed global protein translation in human-induced pluripotent stem cell (hiPSC)-derived forebrain neural progenitor cells (NPCs) from four SZ patients relative to six unaffected controls. We report increased total protein levels and protein synthesis, together with two independent sets of quantitative mass spectrometry evidence indicating markedly increased levels of ribosomal and translation initiation and elongation factor proteins, in SZ hiPSC NPCs. We posit that perturbed levels of global protein synthesis in SZ hiPSC NPCs represent a novel post-transcriptional mechanism that might contribute to disease progression.

## Introduction

We, and others, have reprogrammed fibroblasts from schizophrenia (SZ) patients into human-induced pluripotent stem cells (hiPSCs), and subsequently differentiated these hiPSCs into neural progenitor cells (NPCs) and neurons. SZ hiPSC NPCs show evidence of aberrant adherens junctions^1^ and migration,^2^ increased oxidative stress^2, 3, 4^ and perturbed responses to environmental stressors,^5^ whereas SZ hiPSC neurons exhibit diminished neuronal connectivity,^6^ decreased neurite number^6^ reduced synaptic maturation^3, 7, 8^ and aberrant neurotransmitter activity.^9^ The extent to which genetic or epigenetic mechanisms underlie these differences between heterogeneous SZ patients remains unclear.

Epigenetic mechanisms have been broadly implicated in psychiatric disease (reviewed by Millan,^10^ Mehler^11^ and Tsankova *et al.*^12^) and many global regulators of gene transcription and translation have now been shown to alter the pathophysiology of SZ. Mutations in MECP2, a DNA methyl-binding protein involved in repressive chromatin remodeling and the most common cause of Rett syndrome, have now been linked to SZ.^13^ Target proteins of fragile X mental retardation protein, the product of the *FMR1* gene involved in Fragile X syndrome that acts as a translational repressor by binding to messenger RNA (mRNA), have also been repeatedly associated with SZ.^14, 15, 16^ Furthermore, CYFIP1, a binding partner of fragile X mental retardation protein, is deleted in the SZ-associated copy-number variant at 15q11.2 (refs. 17, 18) has also been linked to SZ. CYFIP1 activity regulates both synaptic protein translation and synaptic actin remodeling in 15q11.2 patients,^19^ resulting in deficits in cell migration, apical polarity and adherens junctions specifically in hiPSC NPCs derived from 15q11.2 patients.^1^ Similarly, protein levels of another FMR1-interacting protein, CYFIP2, were also perturbed in the anterior cingulate cortex of 20 SZ patients.^20^

With so many diverse genetic mechanisms potentially contributing to aberrant gene transcription and protein translation in SZ, we asked whether we could detect aberrant global protein synthesis in SZ hiPSC NPCs. We found evidence for increased total protein levels and increased global translation, in conjunction with increased levels of translation initiation and elongation factors and ribosomal proteins.

## Materials and methods

### Description of SZ patients, hiPSC reprogramming and NPC differentiation

All patient and control NPCs were differentiated from hiPSCs reprogrammed with tetracycline-inducible lentiviruses from fibroblasts obtained from Coriell Cell Repository (Camden, NJ, USA) or ATCC (Manassas, VA, USA). In total, NPCs from four SZ patients and six controls were compared;^2, 6^ all available clinical information is included in Supplementary Table 1. Fibroblasts from all repository patients and controls have been recently genotyped by PsychChip and exome sequencing; copy number variation analysis has previously been reported.^6^

NIMH childhood-onset SZ and control fibroblasts were obtained in collaboration with Judith Rapoport. All participants provided written assent/consent with written informed consent from a parent or legal guardian for minors. Fibroblasts were derived at National Institute of Mental Health and de-identified samples were transferred to the Icahn School of Medicine at Mount Sinai. This study was approved by the Institutional Review Boards of The National Institute of Mental Health and the Icahn School of Medicine at Mount Sinai.

Repository HFs (from six controls and four SZ patients, Supplementary Table 1) were transduced with tetracycline-inducible lentiviruses expressing the transcription factors *OCT4*, *SOX2*, *KLF4*, *cMYC* and *LIN28* ^21^ and then split onto mouse embryonic fibroblasts. Cells were switched to HUES media (KO-Dulbecco's Modified Eagle Medium (DMEM) (Life Technologies, Carlsbad, CA, USA), 10% KO-Serum Replacement (Life Technologies), 10% Plasminate (Talecris, St Paul, MN, USA), 1 × Glutamax (Life Technologies), 1 × NEAA (Life Technologies), 1 × 2-mercaptoethanol (Sigma, St Louis, MO, USA) and 20 ng ml^−1^ FGF2 (Life Technologies) and 1 μg ml^−1^ doxycycline (Sigma) was added to HUES media for the first 21–28 days of reprogramming only.^6^

Embryoid bodies were generated from hiPSCs and then transferred to nonadherent plates (Corning, Corning, NY, USA). Colonies were maintained in suspension in N2 media (DMEM/F12 (Life Technologies), 1 × N2 (Life Technologies) for 7 days and then plated onto polyornithine/Laminin-coated plates. Visible rosettes formed within 1 week and were manually dissected and cultured in NPC media (DMEM/F12, 1 × N2, 1 × B27-RA (Life Technologies), 1 μg ml^−1^ Laminin (Life Technologies) and 20 ng ml^−1^ FGF2 (Life Technologies).^6^

### NPC culture

hiPSC forebrain NPCs were derived as described previously.^6^ Forebrain NPCs were maintained at high density, grown on either Matrigel or Poly-Ornithine/Laminin-coated plates in NPC media (DMEM/F12, 1 × N2, 1 × B27-RA (Life Technologies), 1 μg ml^−1^ Laminin (Life Technologies) and 20 ng ml^−1^ FGF2 (Life Technologies) and split ~1:3–1:4 every week with Accutase (EMD Millipore, Billerica, MA, USA). Cells are tested monthly for mycoplasma (Lonza, Basel, Switzerland).

NPC experiments were conducted on passage-matched populations, generally between passages five and ten. Beyond this, NPC lines begin to show reduced ability to differentiate to neurons (instead showing an increased propensity for astrocyte differentiation), or occasionally undergo spontaneous transformation to a highly proliferative cell incapable of neural differentiation. When any NPC line showed evidence of either increased astrocyte differentiation or cellular transformation, it was dropped from subsequent experiments; for this reason, not all NPC lines were analyzed in all independent experiments. For neuron experiments, three control NPC lines and one SZ NPC line failed to survive the full 6-week differentiation experiment and could not be included.

The following concentrations of drugs were used to assess the impact of oxidative stress on protein synthesis in NPCs (Supplementary Figure 2): 3-day treatment with 2 mm valproic acid (Stemgent, Cambridge, MA, USA), 55 μm (1 ×) β-mercaptoethanol (Life Technologies) (delivered with an equivalent volume of dimethyl sulfoxide (DMSO)) or DMSO; 3-h treatment with 0.05 mm H_2_O_2_, 0.1 mm hydrogen peroxide (H_2_O_2_) or an equivalent volume of phosphate-buffered saline (PBS).

### WGCNA analysis

Weighted gene coexpression network analysis (WGCNA) is widely used to identify modules composed of highly coexpressed genes or proteins.^22, 23, 24, 25, 26^ For this study, WGCNA began with a matrix of the Pearson correlations between all the 3145 differentially expressed proteins, then converted the correlation matrix into an adjacency matrix using a power function *f*(*x*)=*x*^*β*. The parameter *β* (=6.5) of the power function was determined in such a way that the resulting adjacency matrix (that is, the weighted coexpression network), is approximately scale-free. To explore the modular structures of the coexpression network, the adjacency matrix was further transformed into a topological overlap matrix^25^ from which the modules of highly coexpressed proteins were identified based on the average linkage hierarchical clustering and the dynamic cut-tree algorithm.^27^ To distinguish between modules, each module was assigned a unique color identifier, with the remaining, poorly connected genes colored gray. Within-module connectivity for a gene (*g)* in a module (**M**) is the sum of the power-transformed correlations between *g* and all the other genes in the same module **M**.

### LC–MS/MS quantitative mass spectrometry

To compare global protein levels in control and SZ hiPSC NPCs (stably transduced with retroviral green fluorescent protein), we used quantitative label-free liquid chromatography–mass spectrometry (LC–MS)/MS analysis. Cells were washed with PBS, scraped, pelleted, snapfrozen and overnight shipped on dry ice to the Cotter laboratory. There, cells were lysed with TEAB (Sigma) containing Complete, Mini, ethylenediaminetetraacetic acid–free protease inhibitors (Sigma). The lysates were manually sheared with a G27 1/2-needle 10 times, incubated on ice for 30 min and centrifuged for 15 min (16 000 *g*, 4 °C). The supernatant was stored at −80 °C and quantified; 50 μg of protein was digested to peptides with trypsin and purified with C18 tips.

Two micrograms of peptides from trypsin-digested proteins from each control (one NPC line each derived from six controls) and SZ (two NPC lines each derived from four SZ patients) hiPSC forebrain NPC line was injected in triplicate on a Thermo Q-Exactive mass spectrometer equipped with a Dionex Ultimate 3000 (RSLCnano) chromatography system (Thermo Fisher Scientific, Waltham, MA, USA). Peptides were separated using a 60-min reverse-phase gradient at a flow rate of 250 nl min^−1^. Survey full-scan MS spectra (300–1600 Da) were acquired in the Q-Exactive with a resolution of 70 000, and the 12 most intense ions from the preview scan were selected for Higher-Energy Collisional-Induced Dissociation.^28^ The average MS1 peak width was between 15 and 30 s, and the scan time ranged between 1 and 1.5 s, where at least 15 data points were acquired per peak for quantitation. Label-free quantification was performed with Max Quant (V1.3.0.2) as described.^28, 29^ Protein and peptide false discovery rates were set to 0.01, and only proteins with at least two peptides (one uniquely assignable to the protein) were considered as reliably identified. Label-free quantification intensity values were used for protein quantification between groups. Statistical analysis was performed in Perseus (V 1.3.0.4; http://141.61.102.17/perseus_doku/), whereby the data were log2 transformed to eliminate distributional skew and improve the normal approximation for validity of *P*-values. Data imputation was used to replace missing values by values from the normal distribution, and data normalization was performed by subtracting the median label-free quantification intensity per case. Student's *t*-test was then applied across the six control and eight SZ NPC lines, to identify proteins differentially expressed between groups at a 5% threshold, and a permutation-based false discovery rate was applied at a 5% threshold. The Database for Annotation, Visualization and Integrated Discovery (DAVID v6.7; http://david.abcc.ncifcrf.gov/) was applied to statistically significant proteins in order to identify enriched biological themes. This is a distinct analysis of a portion of a larger data set (Topol *et al.*, 2015, under revision).

### Protein quantification by a colorimetric assay

Cells were pelleted by centrifugation and resuspended in 1 × radioimmunoprecipitation assay (RIPA) lysis buffer (Sigma) supplemented with protease and phosphatase inhibitor cocktail tablets (Roche, Basel, Switzerland), triturated and sonicated. For analysis of soluble fractions, but not total protein, the samples were then centrifuged at 10 000 *g* for 10 min at 4 °C. Protein quantification was performed using the Bradford protein assay (Bio-Rad, Hercules, CA, USA), as recommended by the manufacturer, relative to a bovine serum albumin standard (NEB, Ipswich, MA, USA) diluted at 2, 1, 0.5, 0.25 and 0.125 ng μl^−1^.

Total protein levels were first normalized to constitutive renilla reporter levels. NPCs were transfected 3–7 days prior to analysis with a lentiviral-renilla reporter. Renilla levels were determined using the Dual-Glo Luciferase Assay System (Promega, Madison, WI, USA) and measured on a FlexStation 3 (Molecular Devices, Sunnyvale, CA, USA). Bradford protein colorimetric assay (Bio-Rad) protein quantification and lentiviral-renilla quantification were measured in parallel plates.

Total protein levels were also normalized to cell number. In early experiments, 8 million NPCs were counted using a hematocytometer, pelleted by centrifugation and resuspended in 1 × RIPA lysis buffer (Sigma) supplemented with protease and phosphatase inhibitor cocktail tablets (Roche, Basel, Switzerland) for the Bradford protein colorimetric assay. In later experiments, total cell number was simply counted prior to cell lysis, and total protein amount was normalized to cell number.

### Protein synthesis assay using Click-iT chemistry

Cultured fibroblasts, hiPSCs and NPCs were washed with PBS, cultured in DMEM without L-methionine for 4 h, and then in DMEM supplemented with 50 μm L-homopropargylglycine (HPG) for an additional 1 h. HPG content was detected according to the manufacturer's protocol (‘Click-IT', Life Technologies). Briefly, cells were dissociated using Accutase (Millipore), fixed with 4% paraformaldehyde in PBS for 15 min, permeabilized with 0.25% Triton X-100 in PBS for 15 min, washed once with 3% bovine serum albumin in PBS and incubated in Click-IT reaction cocktail (1 × Click-IT reaction buffer and buffer additive, 2 mm CuSO4, 3 μm Alexa Fluor 488-Azide) overnight at 4 °C in the dark. Cells were washed with PBS and then fluorescence was measured by fluorescence-activated cell sorting (FACS) using a BD FACSCanto II (Becton Dickinson, Franklin Lakes, NJ, USA). The median fluorescent intensity and percentage of HPG-positive cells, relative to a negative (no HPG) control, was calculated using FlowJo (FlowJo, Ashland, OR, USA) software. In addition, an estimate of cell size (median forward scatter) was calculated from the FACS data. Owing to the strict time course of L-methionine and HPG incubation, a limited number of replicates could be run in each trial; for our largest experiments (Figure 3c, and SI Figure 1cd) sampling was done in batches and results were normalized across independent tests (no individual experiment alone reached statistical significance).

### mTOR assay

Enzyme-linked immunosorbent assay kit (Abcam, Cambridge, UK) was used, as directed by the manufacturer, to measure baseline mammalian target of rapamycin (mTOR) pSer2448 levels in NPC lysates, normalized to total protein, in untreated conditions.

### Western blot analysis

Cells were isolated, suspended in 1 × RIPA lyses buffer (Sigma) supplemented with Complete Protease Inhibitor tablets (1 per 50 ml) (Roche) and PhosSTOP phosphatase inhibitor tablets (1 per 10 ml), sonicated and then centrifuged at 14 000 *g* for 30 min at 4 °C. Seventy microgram of total protein was separated on 4–20% SDS-polyacrylamide gel (Bio-Rad) and transferred to a nitrocellulose membrane (Bio-Rad). The membrane was blocked with 4% bovine serum albumin (Sigma) in 1.0% Tween (Sigma) in PBS and probed with primary antibodies against mTOR pSer2448 (1:10 000; Abcam) and glyceraldehyde 3-phosphate dehydrogenase (1:10 000; Ambion, Life Technologies) overnight at 4 °C, then with alkaline phosphatase-conjugated secondary antibodies (Sigma) for 2 h at room temperature. The membrane was visualized using 1-step NBT/BCIP (Thermo Fisher Scientific). Imaging was done using a Bio-Rad Gel Doc Imaging System; the membrane was cut during imaging. Semi-quantitative analysis was done with NIH ImageJ (http://imagej.nih.gov/ij/), which was used to compare the density (intensity) of bands.

### Automated image analysis for cell size

At 24 h after plating, NPCs were fixed in 4% paraformaldehyde in PBS at 4 °C for 10 min. NPCs were permeabilized at room temperature for 15 min in 1.0% Triton in PBS, blocked in 5% donkey serum with 0.1% Triton at room temperature for 30 min, and then mouse anti-human NESTIN (Millipore), 1:200 and rabbit anti-βIII-TUBULIN (Covance, Princeton, NJ, USA), 1:200, were incubated in blocking solution overnight at 4 °C. Secondary antibodies were Alexa donkey 555 anti-mouse (Life Technologies) and Alexa donkey 488 anti-rabbit (Life Technologies); all were used at 1:200. To visualize nuclei, slides were stained with 0.5 μg ml^−1^ DAPI (4′,6-diamidino-2-phenylindole) and then mounted with Vectashield (Vector Labs, Burlingame, CA, USA). Images were acquired using the × 10 objective of an Olympus IX51 Fluorescence Microscope (Olympus Corporation of the Americas, Center Valley, PA, USA).

Using the Open-Source Computer Vision package OpenCV version 2.4.9 for Linux (http://opencv.org/), we modified a set of high-content image analysis approaches^30, 31^ that allowed us to do the folllowing (1) extract and enhance each image, (2) combine and compare multi-channels to segment and classify image features (nuclei, soma and dendrites), (3) quantify each classified feature type per image, (4) generate a matrix of all quantified features for each three-channel image set, (5) numerical analysis of ellipse-approximated diameters, oblongness, each classified supervised statistical analyses (analysis of variance per affected/unaffected, cell line and agent/treatment to identify significant morphometric features, including cell diameter and aspect ratio). The script carried out the following steps: (A) each channel's pixel information was normalized and then enhanced. The blue (or DAPI channel) was enhanced using concepts borrowed from high-dynamic-range imaging technique to improve the contour sharpness of the DAPI nuclei. Several band-pass filters were distributed over a range to overcome the problem of non-uniform noise in the DAPI channel. The green (green fluorescent protein) and red channels were enhanced using high-pass filters. (B) The enhanced DAPI channel was segmented and sieved using certain acceptance parameters to obtain the list of DAPI contours. Significant presence of the green or red channel around the DAPI nuclei was used as a metric for green/red fluorescent protein classification. The soma contour was obtained using an annular mask centered around the DAPI nucleus and the internal boundaries for red or green channels were marked out. (C) The soma contours were then fed through several types of metric functions to generate a feature matrix that can be used in step 5. The metrics calculated are (a) count for each cell type, (b) effective cell diameter (mean and s.d. per cell type per image) and (c) cell's aspect ratio (mean and s.d. per cell type per image).

### High-content imaging analysis for cell size

NPCs were seeded onto a Matrigel-coated black plate, clear-bottom 96-well plate (Corning 3603), across a range of cell densities. At 24 h after plating, NPCs were fixed in 4% paraformaldehyde in PBS at 4 °C for 10 min. NPCs were permeabilized at room temperature for 15 min in 1.0% Triton in PBS, blocked in 5% donkey serum with 0.1% Triton at room temperature for 30 min and mouse anti-human NESTIN (Millipore), 1:200 and 200 units per ml phalloidin-568 (Life Technologies) were incubated overnight at 4 °C. Secondary antibodies were Alexa donkey 488 anti-mouse (Life Technologies) at 1:200. To visualize nuclei, slides were stained with 0.5 μg ml^−1^ DAPI (4′,6-diamidino-2-phenylindole). High-content 96-well imaging (× 20 air objective) of NESTIN (red), F-ACTIN (phalloidin, green) and DAPI-stained NPCs was performed using an ImageXpress Ultra (confocal). Images were captured (4 images per well, 12 wells per NPC line, 16 total NPC lines (from six controls and four SZ patients)) and cell segmentation analysis was completed using MetaXpress (Molecular Devices). Data were exported using AcuityXpress (Molecular Devices). Density-matched wells were manually identified; total area of F-ACTIN-positive cells was compared.

### Neurosphere migration assay

Radial neurosphere migration was measured as the previously described migration;^2^ NPCs were dissociated with accutase, cultured for 48 h in nonadherent plates to generate neurospheres, which were manually picked into matrigel matrix (0.5 mg Matrigel was plated in cold NPC media on a 96-well plate 1 h prior to neurosphere plating; following neurosphere picking, additional Matrigel was added (0.5 mg in cold NPC media per 96-well plate). Average radial migration from each neurosphere was measured using ImageJ. Migrating neurospheres were treated for 48 h with either 100 nm rapamycin or a DMSO control.

### Statistical analysis

For phenotypic analysis, statistical analysis was performed using JMP (Cary, NC, USA). Box–Cox transformation of raw data was performed to correct non-normal distribution of the data and residuals. Improvements were assessed by Shapiro–Wilk *W*-test of the transformed data and residuals. Means were compared within diagnosis by one-way analysis using both the Student's *T-*test and Tukey–Kramer honest significant difference test. Finally, a nested analysis of values for individual patients was performed using standard least squares analysis comparing means for all pairs using Student's T test for specific pairs and Tukey–Kramer honest significant difference test for multiple comparisons. Student's *T-*tests were used to test statistical differences between control and SZ LC–MS/MS analyses, with subsequent correction for multiple hypotheses done by Bonferroni methods, as noted. In all figures, except Figure 3c, and SI Figure 1cd, data presented are from a single representative experimental replicate where pooled data represent biological replicate samples within a given experiment: error bars represent s.e. *P<0.05, **P<0.01, ***P<0.001.

## Results

### SILAC quantitative MS identifies increased ribosomal protein levels in SZ hiPSC NPCs

We previously reported applying quantitative large-scale proteomic methods in order to identify quantitative differences in proteome-wide protein levels between control and SZ NPCs.^2^ To measure the relative abundance of thousands of proteins in discovery mode, we used stable isotope labeling by amino acids in cell culture (SILAC), in conjunction with multidimensional protein identification technology, to identify proteins that were up- or downregulated in four pairwise comparisons (consisting of five ‘heavy'/five ‘light' quantitative LCLC–MS/MS analysis runs for each comparison) between gender-matched SZ (P1 and P3) and control (C1, C3 and C6) hiPSC NPCs. Pairwise SILAC comparisons of control and SZ hiPSC NPCs identified changes in cellular adhesion and oxidative stress pathways.^2^

**Figure 1:**
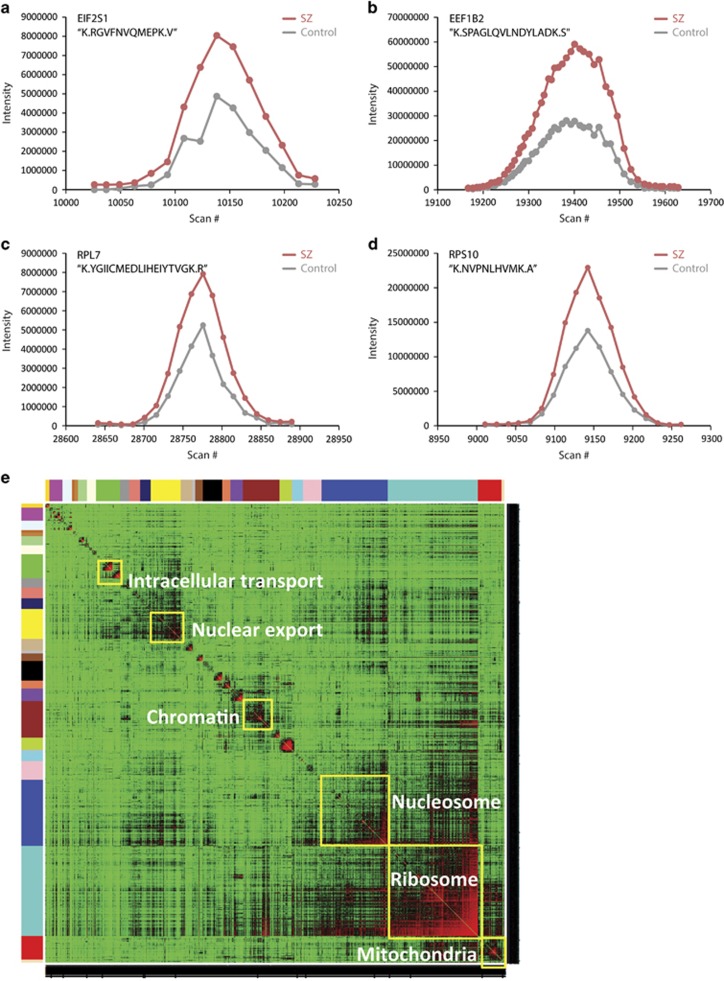
Increased translational machinery in SZ hiPSC NPCs. (**a**–**d**) SILAC MS chromatograms from comparisons of control and SZ NPCs. Representative reconstructed MS1 chromatograms (MS1) spectra from Census for a representative translation initiation factor (EIF2S1) (**a**), elongation factor (EEF1B2) (**b**) and ribosomal proteins (RPL7 (**c**), RPS10 (**d**)) showing significantly increased levels in the SZ (‘light'—red) relative to control (‘heavy'—gray) samples followed by pairwise (C1 × S1A) SILAC mass spectrometry. (**e**) Weighted gene coexpression network analysis of the SILAC and label-free protein coexpression network and corresponding protein modules. A topological overlap matrix of the gene coexpression network of the 9238 proteins altered in at least one of four pairwise SILAC comparisons (C1 × P1A, C1 × P1B, C3 × P3 and C6 × P3) and the 302 proteins altered in the label-free LC–MS/MS experiment, identified 26 protein modules, with sizes ranging from 18 to 608 proteins. The four largest modules (turquoise, blue, brown and yellow) were enriched for proteins involved in ribosome/RNA binding (Bonferroni-corrected *P*=1.6e−14, 3.5-fold), nucleosome (Bonferroni-corrected *P*=1.7e−08, 8.5-fold), chromatin (Bonferroni-corrected *P*=0.16, 5.7-fold) and nucleocytoplasmic transport (Bonferroni-corrected *P*=1.7e−2, 5.8-fold), respectively. hiPSC, human-induced pluripotent stem cell; MS, mass spectrometry; NPC, neural progenitor cell; SILAC, stable isotope labeling by amino acids in cell culture; SZ, schizophrenia.

We completed a focused meta-analysis of this data set, considering only ribosomal proteins and translation (elongation and initiation) factors significantly altered across four independent pairwise SILAC quantitative LC–MS/MS comparisons of control and SZ NPCs (partial list in Table 1, Figures 1a and d). We found that of the 248 significantly perturbed ribosomal proteins and translation initiation factors identified, 235 showed increased abundances (95%), whereas globally 6354 of 9238 (69%) of altered total proteins in SZ hiPSC NPCs were increased. Even though globally more proteins are increased than decreased in SZ hiPSC NPCs, this represents a 1.4-fold over-representation of translation proteins (Fisher's exact test, *P*=1.05e−24).

### Label-free MS also identifies increased ribosomal protein levels in SZ hiPSC NPCs

Although SILAC mass spectrometry methods are extremely sensitive and robust, owing to technical constraints and pairwise analysis, only a very limited number of samples can be compared. Therefore, we confirmed our *post hoc* SILAC analysis using semi-quantitative label-free MS (LC–MS/MS) to generate a quantitative comparison of protein abundances across a substantially larger number of NPC samples from control (one NPC line each derived from six controls) and SZ hiPSC forebrain NPCs (two NPC lines each derived from four SZ patients). Technical triplicates were run and analyzed for each NPC line; data were successfully extracted from 43 of the 45 samples. Of the 2562 proteins detected, our analysis identified 302 proteins that were significantly (*P*<0.05) altered in SZ hiPSC forebrain NPCs relative to control hiPSC forebrain (Supplementary Table 2). We found that of the 34 significantly perturbed ribosomal proteins and eukaryotic translation initiation factors identified, 33 were increased (97%) (Table 2), whereas globally 194 of 302 (64%) of significantly different total proteins in SZ hiPSC NPCs were increased, representing a 1.5-fold over-representation of translation proteins (Fisher's exact test, *P*=2.21e−06).

All proteins with significant changes (*P*<0.05, uncorrected) were submitted to DAVID (http://david.abcc.ncifcrf.gov) for pathway enrichment analysis. Supplementary Table 3 presents the most significantly enriched pathways. The most perturbed pathways include protein synthesis and translation initiation, protein folding and localization as well as RNA processing and binding.

### Network analysis of combined LC–MS/MS data sets

We used WGCNA to identify modules composed of perturbed proteins^22, 23, 24, 25, 26^ (Figure 1e; Supplementary Table 4). By integrating the list of the 9238 proteins altered in at least one of four pairwise SILAC comparisons (C1 × P1A, C1 × P1B, C3 × P3 and C6 × P3) and the 302 proteins altered in the label-free LC–MS/MS experiment, we constructed a coexpression network comprised of 26 coexpressed protein modules, with sizes ranging from 18 to 608 proteins. The four largest modules (turquoise, blue, brown and yellow) were enriched for proteins involved in ribosome/RNA binding (Bonferroni-corrected *P*=1.6e−14, 3.5-fold), nucleosome (Bonferroni-corrected *P*=1.7e–08, 8.5-fold), chromatin (Bonferroni-corrected *P*=0.16, 5.7-fold) and nucleocytoplasmic transport (Bonferroni-corrected *P*=1.7e−2, 5.8-fold), respectively. The turquoise module is the primary driver of protein synthesis; the hub genes of the turquoise module include *RPL32*, *PTGES3*, *HINT2*, *CCT6A* and *RPL37A*. The membership (indicated by the column 'module') of all the proteins and the hubness (indicated by the column 'within-module connectivity') can be found in Supplementary Table 5.

Interestingly, many genes associated with SZ^32^ fall into some coexpressed protein modules. For example, the turquoise module harbors most SZ-associated genes including *ALDOA*, *INA*, *CKAP5*, *CTNNA1* and *HSPD1* (enrichment is not significant). Eleven other modules also include at least one SZ-associated gene and five other modules have one SZ-associated gene. Therefore, this protein coexpression network can be used to perform functional analysis of SZ-associated genes.

### Increased total protein in SZ hiPSC NPCs but not SZ hiPSC neurons

**Figure 2:**
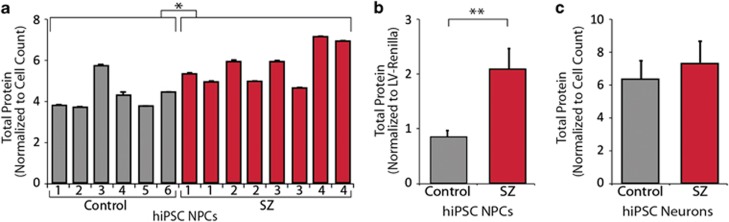
Increased total protein content in SZ hiPSC forebrain NPCs. (**a**) Total protein level of 8 million hiPSC NPCs, quantified by the Bradford protein assay, revealing variation in total protein between individual NPC lines generated from each individual. (**b**) Total protein, quantified by the Bradford protein assay and normalized to constitutive lentiviral luciferase expression. (**c**) Total protein level per million hiPSC neurons, by the Bradford protein assay, averaged by diagnosis. Error bars are s.e. *P<0.05, **P<0.01. hiPSC, human-induced pluripotent stem cell; NPC, neural progenitor cell; SZ, schizophrenia.

Our global MS analyses indicated increased levels of ribosomal proteins and elongation factors; therefore, we used two independent strategies to ask whether in NPCs derived from SZ hiPSCs, there were increased total protein levels. Total protein levels were compared using the Bio-Rad protein colorimetric assay and normalized to cell number (1.2-fold increase, *P*<0.022) (Figure 2a) or renilla fluorescence (following transduction with a constitutive lentiviral reporter) (Figure 2b) (2.5-fold increase, *P*<0.0001). Results were replicated three times normalized to luciferase; each time, we observed significantly increased protein levels in SZ hiPSC NPCs, although there was marked inter-patient variability. Although all experiments described in this report were conducted on passage-matched NPCs (generally between passages 5 and 10, when NPCs most robustly differentiate to neurons), we also directly contrasted lower passage (passages 7–10) and high passage (passages 13–17) NPCs from the same lines, in parallel; we caution that effects are most robust at lower passage. Finally, in matched neurons differentiated from these SZ hiPSC NPCs, total protein levels were compared using the Bio-Rad protein colorimetric assay and normalized to cell number; we did not observe a significant increase in total protein levels in SZ hiPSC neurons (*P*=0.56) (Figure 2c).

Given that we have observed increased ribosomal and translation factor protein levels, concomitant with increased total protein levels, we next asked whether there were changes in global protein synthesis in SZ hiPSC NPCs.

### Global protein synthesis is significantly increased in SZ hiPSC NPCs

**Figure 3:**
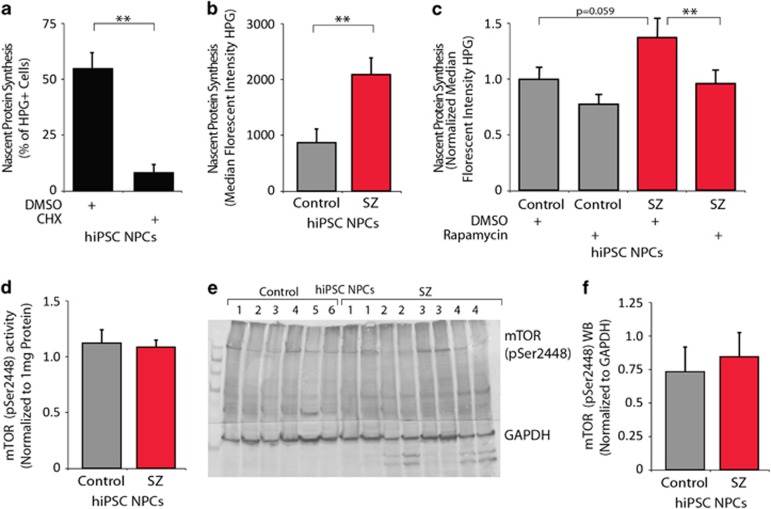
Increased global protein synthesis in SZ hiPSC forebrain NPCs. (**a**) The FACS-based Click-iT L-homopropargylglycine (HPG) assay for nascent protein synthesis shows markedly reduced translation when hiPSC NPCs are treated with 100 μg ml^−1^ CHX. (**b**) Nascent protein synthesis of control and SZ hiPSC NPCs, quantified by a FACS-based HPG assay, averaged by diagnosis. (**c**) Nascent protein synthesis of control and SZ hiPSC NPCs, normalized across four independent experiments, quantified by the FACS-based HPG assay, following treatment with 100 nm rapamycin. (**d**) Enzyme-linked immunosorbent assay measurements of mammalian target of rapamycin (mTOR) pSer2448 levels in control and SZ hiPSC NPC lysates, normalized to protein concentration. (**e**) Western blot analysis of mTOR pSer2448 (~280 kDa) and GAPDH (~40 kDa) levels in control and SZ hiPSC NPC lysates. (**f**) Semi-quantitative analysis of western blot band intensity of mTOR pSer2448, normalized to GAPDH levels, in control and SZ hiPSC NPC lysates. Error bars are s.e. **P<0.01. CHX, cycloheximide; DMSO, dimethyl sulfoxide; FACS, fluorescence-activated cell sorting; GAPDH, glyceraldehyde-3-phosphate dehydrogenase; hiPSC, human-induced pluripotent stem cell; mTOR, mammalian target of rapamycin; NPC, neural progenitor cell; SZ, schizophrenia.

Because our two MS analyses specifically identified significant increases in ribosomal proteins and translational machinery, we hypothesized that increased total protein levels reflect biological differences in the amount of translation in SZ hiPSC NPCs, rather than differences in protein stability, turnover or degradation. To test this, we quantified global protein synthesis rates using a click chemistry approach. This assay measures the incorporation of a methionine analog containing an alkyne moiety (HPG) during active protein synthesis. Addition of the Alexa Fluor 488 azide leads to a chemoselective ligation between the green fluorescent azide and the alkyne, allowing the modified proteins to be quantified by FACS. We first validated the assay by confirming that protein translation in hiPSC NPCs was significantly reduced following treatment with cycloheximide (Figure 3a). Similar to our comparisons of total protein levels, when comparing median fluorescent intensity of HPG-assayed hiPSC NPCs, we observed significantly increased global protein synthesis in SZ hiPSC NPCs (Figure 3b) (2.4-fold, *P*<0.005).

Because treatment of mammalian cells with rapamycin selectively suppresses translation of mRNAs containing an oligopyrimidine tract at their transcriptional start, most notably mRNAs encoding ribosomal proteins and elongation factors,^33, 34, 35^ we tested the effect of rapamycin on control and SZ hiPSC NPCs. When median HPG fluorescence of four independent experiments (normalized to the average median HPG fluorescence of DMSO-treated control hiPSC NPCs) were combined, treatment of SZ hiPSCs with 100 nm rapamycin resulted in a −1.4-fold change in global protein synthesis (*P*<0.007)(Figure 3c). Rapamycin-induced rescue of increased protein translation need not imply increased baseline mTOR activity in SZ hiPSC NPCs; an enzyme-linked immunosorbent assay for active mTOR (phosphorylated at Ser2448), normalized to protein concentration, failed to detect any significant differences in active mTOR between control and SZ hiPSC NPCs in untreated (baseline) conditions (*P*=0.77) (Figure 3d). This was confirmed by western blot analysis of mTOR pSer2448 (~280 kDa) and glyceraldehyde 3-phosphate dehydrogenase (~40 kDa) levels (Figures 3e and f).

To determine whether perturbed protein synthesis is restricted to NPCs, we quantified global protein synthesis rates in hiPSCs (Supplementary Figure 1A) and primary fibroblasts (Supplementary Figure 1B) from the same six control and four SZ individuals. We did not observe increased protein synthesis in SZ hiPSCs, which may reflect that pluripotent stem cells are thought to have low mRNA translation and protein accumulation.^36^ Unexpectedly, we detected differences in global protein synthesis in SZ fibroblasts (1.7-fold, *P*<0.01), suggesting that this phenotype may not be restricted to NPCs. However, when we tested global protein synthesis across a second cohort of fibroblasts, derived from 10 control and 12 childhood-onset SZ patients (defined by clinical onset between the ages of 6 and 12 years^37, 38, 39^), we observed only a trend of increased protein synthesis (1.1-fold, *P*=0.0778) (Supplementary Figure 1C); protein synthesis was not increased in every patient and there was a high degree of inter-individual variation, even among controls.

### No evidence of increased cell size in SZ hiPSC NPCs

**Figure 4:**
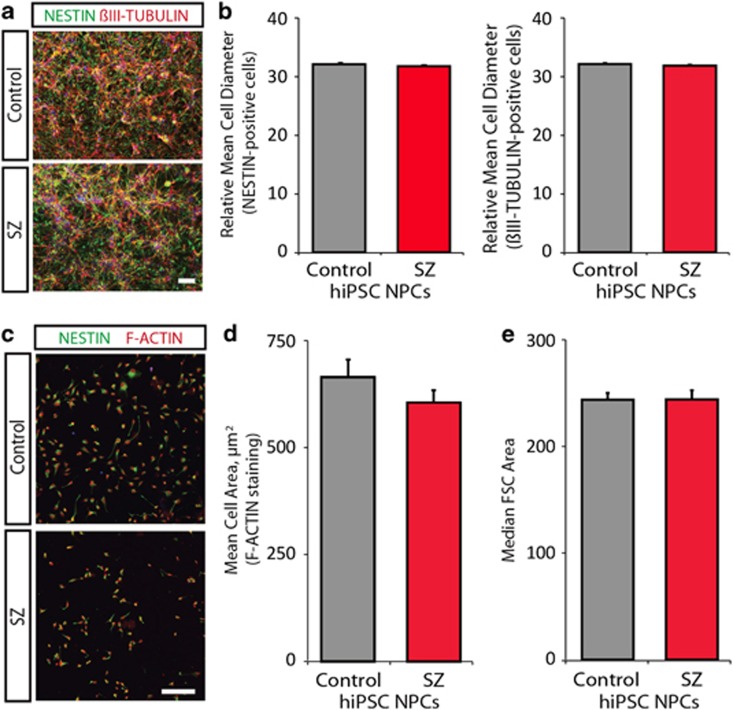
No apparent difference in cell size between control and SZ hiPSC forebrain NPCs. (**a**) Representative images of control and SZ NPCs stained with the NPC marker NESTIN (green) and the neuronal marker βIII-TUBULIN (red). DAPI-stained nuclei (blue). Scale bar, 100 μm. (**b**) Automated image analyses of the mean cell diameter and mean cell aspect ratio of both NESTIN-positive (136 090 DAPI-positive cells quantified across 71 control and 81 SZ NPC images; 0.99-fold increase in cell diameter, *P*<0.0001; 1.00-fold increase in cell aspect ratio, *P*<0.0001; *P*<0.83) and TUJ1-positive (68 685 DAPI-positive cells quantified across 36 control and 41 images; 0.99-fold increase in cell diameter, *P*<0.0001; 1.00-fold increase in cell aspect ratio, *P*<0.32) control and SZ hiPSC NPCs. (**c**) Representative high-content images of control and SZ NPCs stained with the NPC marker NESTIN (green) and the F-ACTIN (phalloidin, red). DAPI-stained nuclei (blue). Scale bar, 100 μm. (**d**) MetaXpress automated cell segmentation and size analysis to calculate the mean cell area of F-ACTIN-positive (5985 F-ACTIN-positive cells quantified across 76 control and 176 SZ NPC images; 0.91-fold increase in cell diameter, *P*<0.27) control and SZ hiPSC NPCs. (**e**) Fluorescence-activated cell sorting analysis for median FSC of control and SZ hiPSC NPCs. Error bars are s.e. DAPI, 4′,6-diamidino-2-phenylindole; FSC, forward scatter; hiPSC, human-induced pluripotent stem cell; NPC, neural progenitor cell; SZ, schizophrenia.

Increased protein synthesis in SZ hiPSC NPCs could be either a cause or consequence of increased cell size. To test this, we estimated the size of control and SZ hiPSC NPCs by microscopy, as well as by FACS. First, in a monolayer, NPCs were stained with NESTIN and βIII-TUBULIN, markers of NPCs and neurons, respectively, within the population (Figure 4a). Automated image analyses found no meaningful differences between the mean cell diameter or mean cell aspect ratio of both NESTIN-positive (136 090 DAPI-positive cells quantified across 71 control and 81 SZ NPC images; 0.99-fold increase in cell diameter, *P*<0.0001; 1.00-fold increase in cell aspect ratio, *P*<0.0001; *P*<0.83) and TUJ1-positive (68 685 DAPI-positive cells quantified across 36 control and 41 images; 0.99-fold increase in cell diameter, *P*<0.0001; 1.00-fold increase in cell aspect ratio, *P*<0.32) control and SZ hiPSC NPCs (Figure 4b). However, this analysis was complicated by the high density of the cells in the images, as well as because neither NESTIN nor βIII-TUBULIN labels the entire cytoskeleton, as revealed by F-ACTIN staining. Second, high-content image analysis was repeated in 96-well format, to permit precise matching of cell densities between images. High-content images of control and SZ NPCs stained with the NPC marker NESTIN (green) and F-ACTIN (phalloidin, red) permitted staining of the area of each cell (Figure 4c). MetaXpress automated cell segmentation of DAPI-positive cells based on F-ACTIN staining, in order to calculate the mean cell area of F-ACTIN-positive NPCs (5985 F-ACTIN-positive cells were quantified across 76 control and 176 SZ NPC images; 0.91-fold increase in cell diameter, *P*<0.27 control and SZ hiPSC NPCs (Figure 4d). Third, FACS analysis for median forward scatter of control and SZ hiPSC NPCs also detected no significant difference in cell size (Figure 4e).

Therefore, by image-based and FACS-based experimental methods, it seems unlikely that increased protein synthesis in SZ hiPSC NPCs can be explained by increased cell size in SZ hiPSC NPCs.

### Rapamycin treatment does not ameliorate aberrant migration in SZ hiPSC NPCs

**Figure 5:**
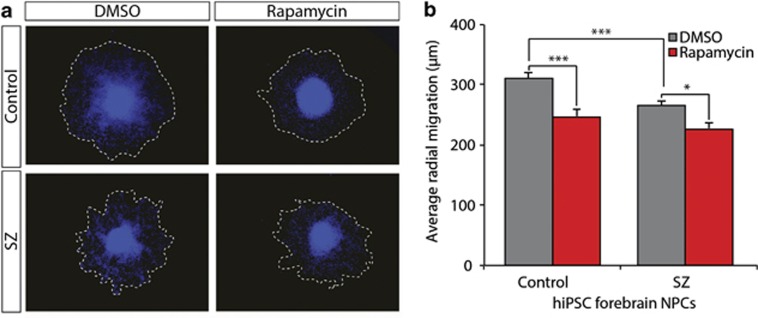
Rapamycin treatment is not sufficient to rescue aberrant migration in SZ hiPSC forebrain NPCs. (**a**) Representative images of control and SZ neurosphere outgrowth, with and without 48-h treatment with 100 nm rapamycin. (**b**) Decreased overall neurosphere outgrowth with 48-h treatment with 100 nm rapamycin. Error bars are s.e. *P < 0.05, ***P<0.001. DMSO, dimethyl sulfoxide; hiPSC, human-induced pluripotent stem cell; NPC, neural progenitor cell; SZ, schizophrenia.

We previously reported that SZ hiPSC NPCs show reduced radial migration in a neurosphere assay.^2^ To test whether aberrant migration can be rescued by inhibiting protein synthesis, neurospheres were cultured with 100 nm rapamycin, or a DMSO control, for the duration of the 48-h neurosphere migration assay; 102 SZ neurospheres were analyzed relative to 114 control neurospheres. Although 100 nm rapamycin treatment was sufficient to reduce translation in SZ hiPSC NPCs (Figure 3d), it did not improve migration in SZ NPCs (Figure 5); in fact, both control (0.79-fold, *P*<0.000001) and SZ (0.85-fold, *P*<0.011) neurospheres showed significantly impaired migration with rapamycin (Figures 5a and b). Consequently, we posit that increased protein synthesis in SZ hiPSC NPCs is unlikely to underlie the aberrant migration that we previously reported in these SZ hiPSC NPCs.

## Discussion

Here we report increased total protein levels and global protein synthesis in SZ hiPSC NPCs, but not SZ hiPSCs or hiPSC neurons, derived from four SZ patients. It is important to note that that increased protein synthesis holds true as a group effect only; it is insufficient to predict diagnosis of any given individual. Although the molecular mechanism underlying this phenotype across genetically heterogeneous patients remains unknown, two independent MS comparisons (SILAC and label-free LC–MS/MS) of control and SZ hiPSC NPCs found that, either by WGCNA or DAVID analysis, the protein network or pathways that were most perturbed included protein synthesis and RNA processing and binding. Moreover, of the significantly perturbed ribosomal proteins and translation initiation factors identified, 95 and 97%, respectively, were elevated in these two MS analyses, suggesting that increased protein synthesis, at least in part, is a direct result of increased translational machinery in SZ hiPSC NPCs. Consistent with our hypothesis of increased ribosomal translation, global protein synthesis in SZ hiPSC NPCs could be restored to control levels by treatment with rapamycin. Given the increased rate of protein synthesis in SZ hiPSC NPCs, we believe that this will be a valuable model for studying the global translational machinery contributing to neural dysfunction in SZ, much as studies of human embryonic stem cell-derived Rett Syndrome (RTT) neurons have revealed global transcriptional and translational repression^40^ in this monogenic model of autism spectrum disorder.

Protein synthesis is differentially regulated between cells types, including stem cells, in order to establish and maintain differences in cell identity and function (reviewed in Buszczak *et al.*^41^). On the basis of our data, and that of English *et al.*,^42^ aberrant regulation of protein synthesis in SZ appears to be cell-type dependent. Although we observed increased protein synthesis in SZ hiPSC NPCs (a neural cell type most resembling fetal forebrain tissue^2^), English *et al.* report decreased protein synthesis in olfactory neural stem (ONS) cells derived from nine SZ patients and nine controls. These distinct but complementary observations may indicate that protein translation is affected by neural subtype identity and developmental state^43^ and/or differences in neuronal maturation and activity;^44, 45^ this would not be inconsistent with the observation that reduced global translation is specific to RTT embryonic stem cell-derived neurons and not detected in RTT embryonic stem cell-derived NPCs.^40^ Together our data suggest that aberrant translational regulation may contribute to SZ predisposition and/or disease state. Understanding the molecular mechanism(s) contributing to this loss of translational control may lead to new treatments for this devastating disorder.

A number of important experimental limitations may contribute to the conflicting direction of change in protein synthesis between hiPSC NPCs and ONS cells. First, these two studies investigate relatively small patient cohorts, recruited on different criteria. Until both studies have been replicated across substantially larger cohorts, and functional studies testing the effect of perturbed protein synthesis on synaptic function in human neurons have been conducted, the biological impact of perturbed global protein synthesis to the full SZ disease spectrum cannot be known. Second, these two studies compare *in vitro* expanded and manipulated neural cell types, and may not reflect actual conditions within the human brain; moreover, hiPSC NPC and ONS cells are cultured in different growth media and on different substrates, and both neural populations have been stressed and exposed to conditions, such as high oxygen levels, not present in the human brain. Third, hiPSC NPCs and ONS cells are fundamentally different neural cell types, with different temporal (fetal versus adult) and spatial (forebrain versus olfactory mucosa) patterning, being heterogeneous populations comprised of distinct compositions of neural cell types (excitatory and inhibitory neurons, as well as astrocytes), and with different abilities to generate synaptically active neurons. Nonetheless, we feel that the loss of translational regulation observed across such disparate approaches is meaningful. To resolve these inconsistencies, future studies must compare many more hiPSC-derived neural cell types (glutamatergic, GABAergic and dopaminergic neurons, as well as astrocytes) across larger cohorts, and should ideally include patient-matched ONS cells derived from the same cohort as well.

Accumulation of proteins is a hallmark of senescence,^46^ which can be initiated by the shortening of telomeres (replicative senescence) or by stress signals,^47^ making it a potential experimental confound to our conclusions. With increasing passage, hiPSC NPCs show increased propensity for gliogenesis and transformation;^48^ however, it is also possible that SZ cells have an increased susceptibility to senescence that could be biasing our results, and we note that although we previously reported no measurable differences in proliferation, neurogenesis or cell death in SZ hiPSC NPCs, we did detect elevated susceptibility to apoptosis^2^ and sub-threshold cellular stresses.^5^ In all experiments described herein, NPCs were passage-matched when evaluated, growing robustly and still capable of efficient neuronal differentiation; nonetheless, we caution that our results may not remain robust with prolonged culture.

A variety of mechanisms, ranging from global transcription,^49^ mRNA stability and degradation,^50^ microRNA gene regulation,^51, 52^ protein folding and stability^53^ to ubiquitination,^54, 55^ can affect total protein levels and have been linked to SZ. Here, we report evidence of increased protein synthesis in SZ hiPSC NPCs. Translational dysregulation in SZ has been previously suggested to explain protein abnormalities contributing to SZ.^56^ A number of biological factors (oxidative stress,^57^ ribosome protein transcription or translation^58^ and mTOR signaling^59^) affect protein synthesis; all of which have been implicated in SZ and could contribute to the mechanism underlying our observations and are discussed below.

Although oxidative stress is one of the best-studied regulators of gene transcription,^60^ there is also a complex translational response to oxidative stress.^57^ SZ hiPSC-based studies have recently reported findings of increased reactive oxygen species,^4^ increased oxidative stress,^2, 3^ increased sensitivity to sub-threshold environmental stresses^2, 5^ and impaired mitochondrial structure and function.^2, 3^ We speculated that increased oxidative stress in SZ hiPSC NPCs could contribute to the perturbations in global protein translation, which we observed in hiPSC NPCs from our four SZ patients; however, treatment with 55 μm β-mercaptoethanol or 2 mm valproic acid (previously reported to reduce increased extra-mitochondrial oxygen consumption in SZ hiPSC neural cells^4^) did not reduce protein synthesis in hiPSC NPCs (Supplementary Figure 2A), and culture with 0.05 mm H_2_O_2_ or 0.1 mm H_2_O_2_ for 3 h did not increase protein synthesis in hiPSC NPCs (Supplementary Figure 2B). Nonetheless, it remains possible that careful analysis at the single-cell level may better link increased oxidative stress, mitochondrial dysfunction, intra-cellular stress response and changes in global protein synthesis.

Ribosome biogenesis involves the coordinated function of over 200 proteins in the synthesis, processing and assembly of ribosomal RNAs with the ribosomal proteins (reviewed in Zaher and Green^58^). Although post-mortem gene expression studies of ribosomal subunit genes can be greatly confounded by post-mortem interval,^61^ a recent post-mortem RNAseq-based comparison of nine male SZ subjects and nine matched non-psychiatric controls clustered the differentially expressed genes into functional groups, generating a network consisting of six functional clusters, one of which was ribosome and translation activity.^49^ In addition, ribosomal DNA transcription in the dorsal raphe nucleus was increased in 9 residual (but not in 8 paranoid) SZ patients, relative to 28 matched controls.^62^ It has been hypothesized that disturbances in ribosomal DNA transcriptional activity contribute to predisposition to psychiatric disease.^63^ Limited numbers of post-mortem brain MS comparisons have been completed on SZ patient brains; however, differences have been reported in ribosomal protein levels,^20^ as well as proteins associated with protein folding and synthesis^64^ and nucleotide and protein metabolism.^65^

In addition to selectively suppressing translation of mRNAs encoding ribosomal proteins and elongation factors,^33, 34, 35^ rapamycin also inhibits mTOR signaling cascade, which regulates the translation of proteins involved in neuronal morphology and synaptic plasticity, and may be disrupted in SZ (reviewed in Gururajan and van den Buuse^66^). Signaling information from a variety of neurotransmitter receptors is integrated by mTOR, and can lead to increased synaptic signaling.^67^ First, both ionotropic (N-methyl-D-aspartate receptor) and metabotropic glutamate receptors initiate mTOR-dependent protein synthesis;^68^ the N-methyl-D-aspartate hypofunction mutant mouse show reduced Akt/mTOR signaling, together with reduced dendritic spines, synaptic plasticity and hippocampal volume.^69^ Second, the SZ-associated gene reelin (*RELN*) induces recruitment of the mTOR upstream activators Akt and PI3K;^70, 71^ moreover, heterozygous RELN^+/−^ mice show reduced dendritic spine density and long-term potentiation; ketamine rescue of this phenotype is prevented by rapamycin.^72^ Third, Disrupted in Schizophrenia-1 (DISC1) interacts directly with AKT–mTOR signaling, and cognitive and affective deficits in DISC1 knockout mice can be rescued by rapamycin treatment.^73, 74^ Fourth, treatment of CYFIP1 overexpressing neural progenitors with rapamycin rescued the morphological abnormalities.^75^ Although we did not detect abnormalities in mTOR signaling in SZ hiPSC NPCs, we cannot discern through which mechanism, translational repression or mTOR inhibition, rapamycin rescued aberrant translation in our SZ hiPSC NPCs.

We postulate that general loss of translational control may either contribute to, or serve as a biomarker of, SZ risk. Although our data suggest that increased protein synthesis may occur in NPCs derived from these four SZ patients, it is critical to note that this study is too small to draw conclusions about the larger SZ patient population; moreover, we have not distinguished whether translational dysregulation is a cause or consequence of disease. This work must now be broadly replicated across diverse SZ patient cohorts, in order to understand how frequently, and to what extent, translational dysregulation occurs in SZ.

## Figures and Tables

**Table 1:** Significantly increased translation factors and ribosomal proteins in SZ hiPSC NPCs observed by SILAC quantitative mass spectroscopy

*SILAC*	*Gene family*	*Protein names*	*Up*	*Down*	*Global up*	*Global down*
P1A × C1					2289	752
	Translation initiation factor	Up: EIF2AK2, EIF2AK4, EIF2B1, EIF2B2, EIF2B3, EIF2S2, EIF3S3, EIF3S5, EIF3S6, EIF3S7, EIF3S8, EIF3S9, EIF3S10, EIF3S12, EIF4A2, EIF4A3, EIF4AB, EIF4G1, EIF4G2, EIF4G3, EIF5, EIF5A, EIF5B	38	0		
		Down:				
	Ribosomal protein	Up: RPL3, RPL4, RPL6, RPL7, RPL8, RPL9, RPL10A, RPL11, RPL12, RPL13, RPL14, RPL15, RPL17, RPL18, RPL18A, RPL21, RPL22L1, RPL23, RPL23A, RPL24, RPL27A, RPL32, RPL36A, RPL37A, RPL38, RPLP0, RPLP1, RPLP2, RPN, RPN2, RPS2, RPS3, RPS6, RPS7, RPS10, RPS11, RPS12, RPS13, RPS17, RPS19, RPS20, RPS21, RPS24, RPS25, RPS27	66	0		
		Down:				
P1B × C1					2401	886
	Translation initiation factor	Up: EIF2AK2, EIF2AK4, EIF2B1, EIF2B2, EIF2B3, EIF2S2, EIF2S3, EIF3S3, EIF3S4, EIF3S5, EIF3S6, EIF3S8, EIF3S9, EIF3S10, EIF3S12, EIF4A2, EIF4A3, EIF4B, EIF4G1, EIF4G2, EIF4G3, EIF5, EIF5A, EIF5B	36	0		
		Down:				
	Translation elongation factor	Up: EEF1A1, EEF1A2, EEF1B2, EEF1E1, EEF1G, EEF2Down:	7	0		
	Ribosomal protein	Up: RPL3, RPL4, RPL6, RPL7, RPL7A, RPL8, RPL9, RPL10A, RPL14, RPL15, RPL17, RPL18, RPL18A, RPL21, RPL22L1, RPL23, RPL26, RPL26L1, RPL27A, RPL32, RPL36A, RPL37A, RPL38, RPLP0 RPLP1, RPLP2, RPLP10, RPS2, RPS3, RPS6, RPS7, RPS10, RPS12, RPS13, RPS17, RPS19, RPS20, RPS21, RPS24, RPS25, RPS27	48	1		
		Down: RPL22				
P3 × C3					1123	366
	Translation initiation factor	Up: EIF2B1, EIF2S2, EIF2S3,EIF3S2, EIF3S4, EIF3S5, EIF3S12, EIF4A3, EIF4E, EIF5, EIF5A	10	4		
		Down: EIF2C1, EIF2C3, EIF2C12				
	Translation elongation factor	Up: EEF1A1, EEF1A2, EEF1GDown:	3	0		
	Ribosomal protein	Up: RPL3, RPL8, RPL9, RPL10A, RPL14, RPL15, RPL17, RPL23, RPL32, RPLP1, RPLP2, RPS2, RPS7, RPS17	14	1		
		Down: RPL22L1				
P3 × C6					541	880
	Translation initiation factor	Up: EIF3S5, EIF4G1, EIF5A	3	4		
		Down: EIF2A, EIF2C1, EIF2C3,ELAV				
	Ribosomal protein	Up: RPA3, RPL21, RPL23A, RPL23AP2, RPL8, RPLP2, RPS3A, RPS29	10	3		
		Down: RPL11, RPL36, RPS12,				
	Total		235	13	6354	2884

Abbreviations: hiPSC, human-induced pluripotent stem cell; NPC, neural progenitor cell; SILAC, stable isotope labeling by amino acids in cell culture; SZ, schizophrenia.

**Table 2:** Significantly increased translation factors and ribosomal proteins in SZ hiPSC NPCs observed by label-free LC–MS/MS quantitative mass spectroscopy

*Gene family*	*Protein names*	*Up*	*Down*	*Global up*	*Global down*
				194	108
Translation initiation factor	Up: EIF1, EIF2S3, EIF3A, EIF3C, EIF3F, EIF4A, EIF4A3, EIF5A, EIF6	9	0		
	Down:				
					
Translation elongation factor	Up: EEF1A1, EEF2	2	0		
	Down:				
					
Ribosomal protein	Up: RPL3, RPL7, RPL7A, RPL10, RPL10A, RPL12, RPL21, RPL22, RPL23, RPL27,RPL30, RPL36, RPLP0, RPLP2, RPS3, RPS8, RPS11,RPS12, RPS17L, RPS25, RPS26, RPSA	22	1		
	Down: PRPS1				
Total		33	1	194	108

Abbreviations: hiPSC, human-induced pluripotent stem cell; LC–MS/MS, liquid chromatography–mass spectrometry/MS; NPC, neural progenitor cell; SZ, schizophrenia.
