# Supplementary figures and images for: Increased abundance of translation machinery in stem cell–derived neural progenitor cells from four schizophrenia patients
Source: Transl Psychiatry. 2015 Oct 20;5(10):e662–. doi: 10.1038/tp.2015.118 (PMC4930118; doi:10.1038/tp.2015.118)

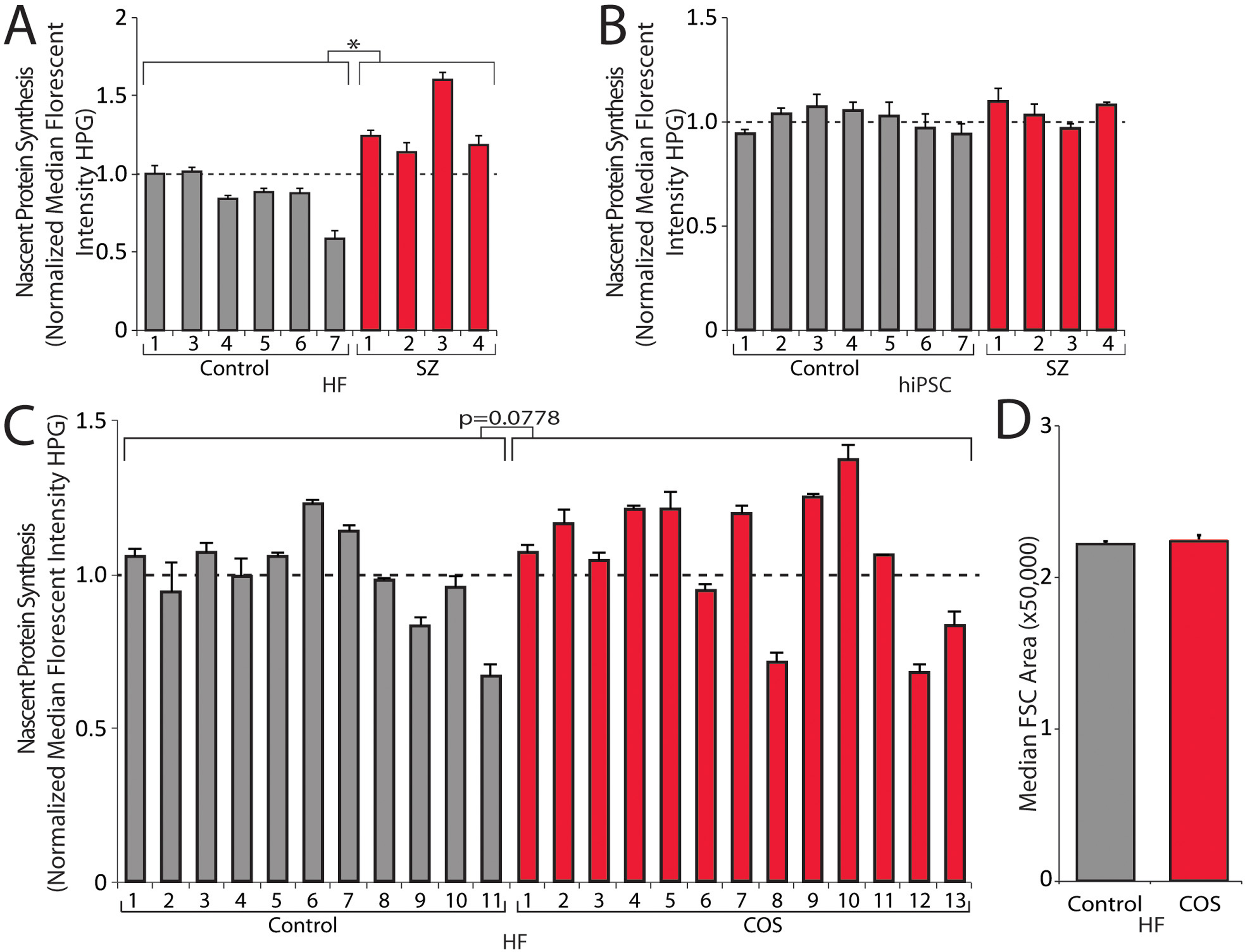

Supplement: Supplementary Figure 1 [file tp2015118x1.tif]

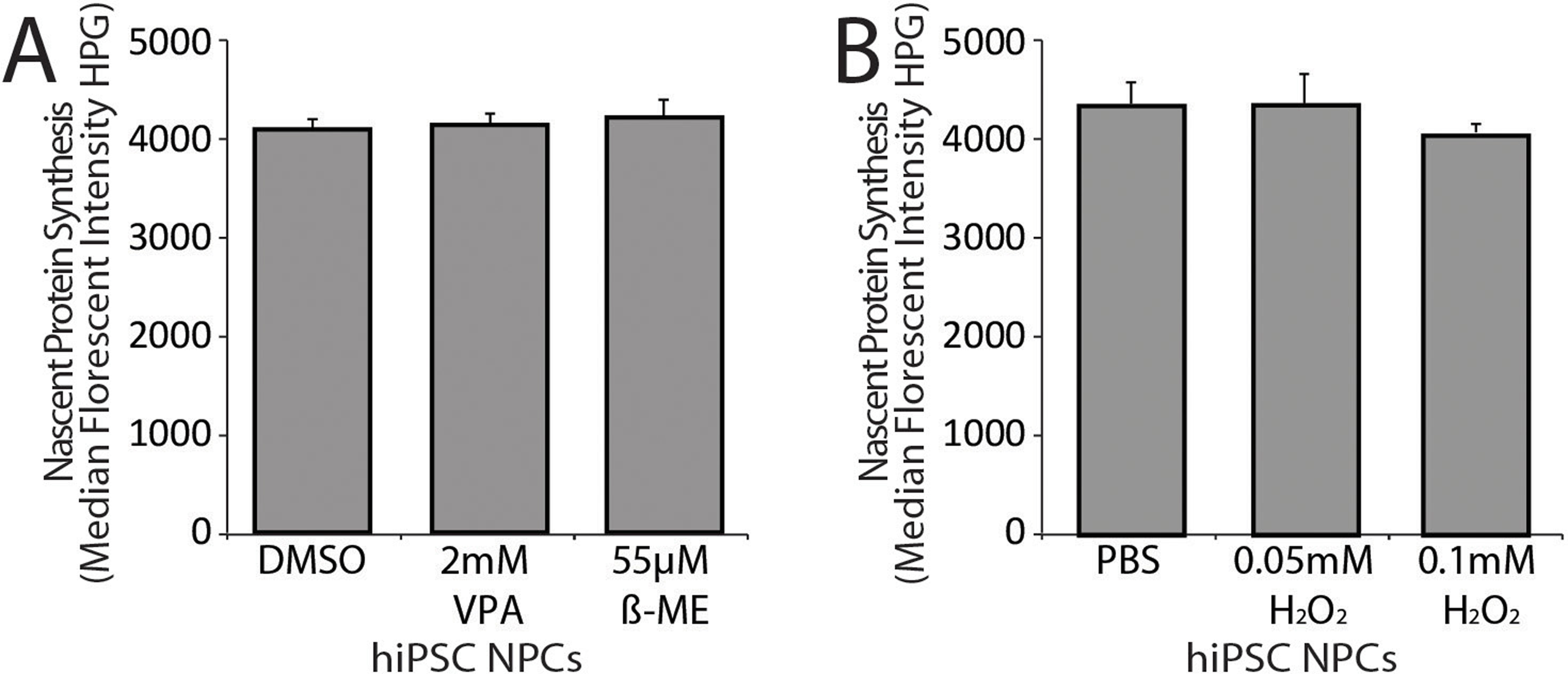

Supplement: Supplementary Figure 2 [file tp2015118x2.tif]
