# Supplementary material for: Increased abundance of translation machinery in stem cell–derived neural progenitor cells from four schizophrenia patients
Source: Transl Psychiatry. 2015 Oct 20;5(10):e662–. doi: 10.1038/tp.2015.118 (PMC4930118; doi:10.1038/tp.2015.118)
Supplement: Supplementary Information [file tp2015118x4.docx]

**SUPPLEMENTARY INFORMATION**

**SI Table 1. Description of clinical information available for control and SZ patients.**

**SI Table 2. Label-free LC MS/MS quantitative analysis of proteins significantly altered in SZ hiPSC forebrain NPCs.**

**SI Table 3. DAVID analysis of biological clusters most enriched in in label-free LC MS/MS dataset from** **SZ hiPSC forebrain NPCs.**

**SI Table 4. Gene Ontology analysis of WGCNA modules derived from SILAC and label-free LC MS/MS quantitative analysis of proteins significantly altered in SZ hiPSC forebrain NPCs.**

**SI Table 5. Module assignment and gene connectivity from the gene coexpression network analysis. Genes in each module are sorted in the descendant order of within-module connectivity.**

**SI Figure 1. Changes in global protein synthesis in SZ fibroblasts but not SZ hiPSCs. A-B**. Nascent protein synthesis of control and SZ fibroblasts (**A**) and hiPSCs (**B**), quantified by FACS-based HPG Assay, averaged by individual. **C**. Nascent protein synthesis in a replication cohort comprised of ten unrelated controls and twelve childhood-onset-SZ cases, quantified by FACS-based HPG Assay, averaged by individual. **D.** FACS analysis for median forward scatter (FSC) of control and COS fibroblasts. Error bars are s.e. *P < 0.05, **P < 0.01, ***P < 0.001.

**SI Figure 2. No apparent effect of manipulating oxidative stress on nascent protein synthesis. A.** Average nascent protein synthesis of two hiPSC NPC lines, quantified by FACS-based HPG Assay, following three-day treatment wither either 2 mM valproic acid (VPA), 55 μM 2-mercaptoethanol (β-ME) (delivered with an equivalent volume of DMSO), or DMSO. **B.** Average nascent protein synthesis of two hiPSC NPC lines, quantified by FACS-based HPG Assay, following three-hour treatment with 0.05 mM H_2_O_2_, 0.1 mM H_2_O_2_, or an equivalent volume of PBS.
